# Supplementary material for: Structural insights into intron catalysis and dynamics during splicing
Source: Nature. 2023 Nov 22;624(7992):682–8. doi: 10.1038/s41586-023-06746-6 (PMC10733145; doi:10.1038/s41586-023-06746-6)
Supplement: Supplementary file 1 — Supplementary Fig. 1: CryoEM sample preparation denaturing gel from Fig. 1d. a, Various splicing conditions were used to obtain samples that contained the prebranching, preligation and postligation complexes. b, An SDS gel showing the protein contents of the same samples in bottom inset of a. Boxed regions indicate the cropped images shown in the main text figure. c, Splicing assay denaturing gels shown in Fig. 2d,h. All four replicates of the gels are shown. Boxed regions indicate the cropped images shown in the main text figure. Supplementary Fig. 2: Splicing gel time courses in the absence or presence of WT maturase protein from Extended Data Fig. 1a,c. Boxed regions indicate the cropped images shown in the Extended Data Fig. 1. [file 41586_2023_6746_MOESM1_ESM.pdf]

nature portfolio

https://doi.org/10.1038/s41586-023-06746-6

Supplementary information

Structural insights into intron catalysis and dynamics during splicing

In the format provided by the
authors and unedited

Supplementary Information Figure S1

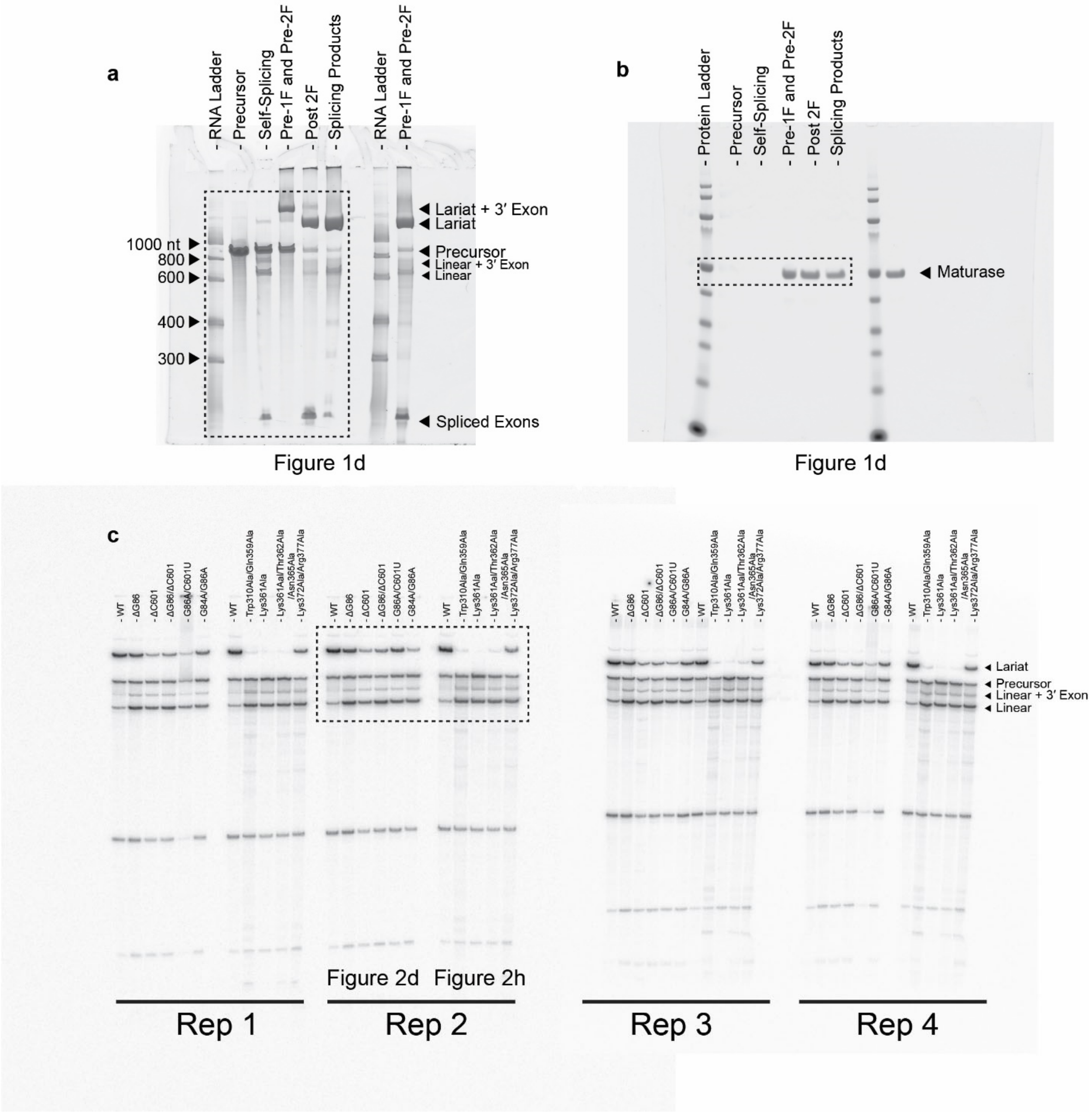

Supplementary Information Figure S2

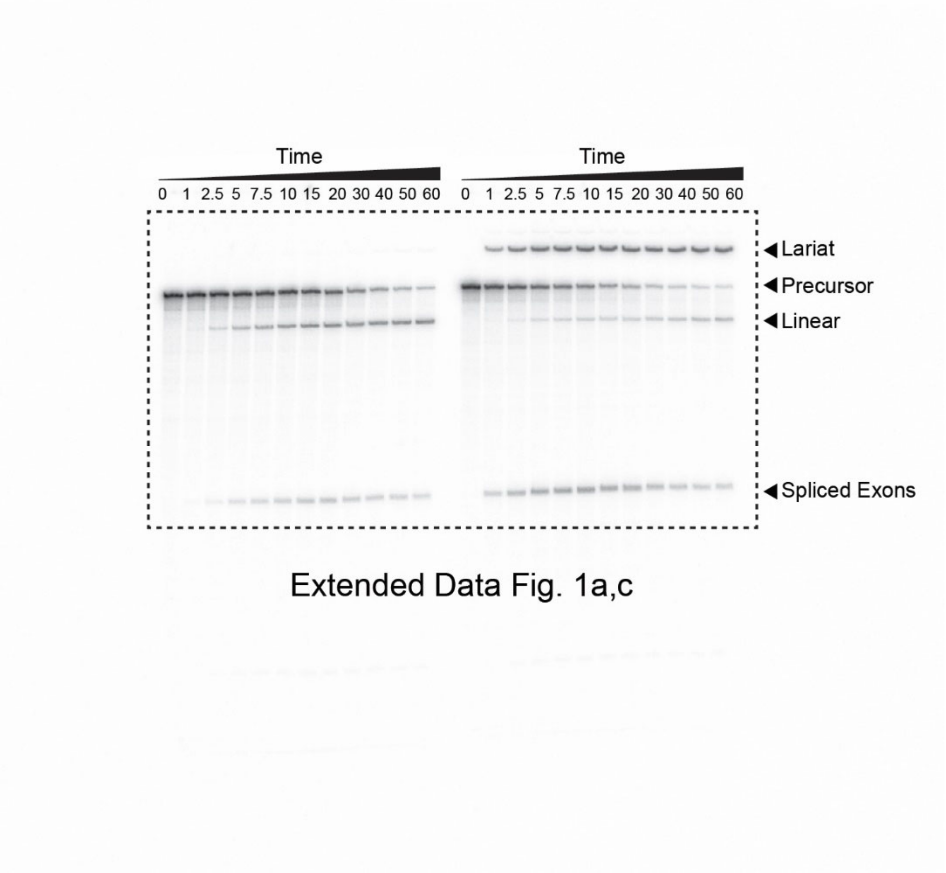

Extended Data Fig. 1a,c
