# Supplementary material for: Structural insights into intron catalysis and dynamics during splicing
Source: Nature. 2023 Nov 22;624(7992):682–8. doi: 10.1038/s41586-023-06746-6 (PMC10733145; doi:10.1038/s41586-023-06746-6)
Supplement: Supplementary file 2 — Reporting Summary [file 41586_2023_6746_MOESM2_ESM.pdf]

nature portfolio

Corresponding author(s): Ling Xu and Anna Marie Pyle

Last updated by author(s): Sep 22, 2023

Reporting Summary

Nature Portfolio wishes to improve the reproducibility of the work that we publish. This form provides structure for consistency and transparency in reporting. For further information on Nature Portfolio policies, see our Editorial Policies and the Editorial Policy Checklist.

Statistics

For all statistical analyses, confirm that the following items are present in the figure legend, table legend, main text, or Methods section.

n/a	Confirmed	
☒	☐	The exact sample size (*n*) for each experimental group/condition, given as a discrete number and unit of measurement
☐	☒	A statement on whether measurements were taken from distinct samples or whether the same sample was measured repeatedly
☒	☐	The statistical test(s) used AND whether they are one- or two-sided<br>*Only common tests should be described solely by name; describe more complex techniques in the Methods section.*
☒	☐	A description of all covariates tested
☒	☐	A description of any assumptions or corrections, such as tests of normality and adjustment for multiple comparisons
☒	☐	A full description of the statistical parameters including central tendency (e.g. means) or other basic estimates (e.g. regression coefficient) AND variation (e.g. standard deviation) or associated estimates of uncertainty (e.g. confidence intervals)
☒	☐	For null hypothesis testing, the test statistic (e.g. *F*, *t*, *r*) with confidence intervals, effect sizes, degrees of freedom and *P* value noted<br>*Give P values as exact values whenever suitable.*
☒	☐	For Bayesian analysis, information on the choice of priors and Markov chain Monte Carlo settings
☒	☐	For hierarchical and complex designs, identification of the appropriate level for tests and full reporting of outcomes
☒	☐	Estimates of effect sizes (e.g. Cohen's *d*, Pearson's *r*), indicating how they were calculated

Our web collection on statistics for biologists contains articles on many of the points above.

Software and code

Policy information about availability of computer code

Data collection	SerialEM v3.9 and v4.0 (cryoEM data were collected on a Thermo Fisher Titan Krios microscope equipped with a Gatan G3 Summit Detector)
Data analysis	All software are publicly accessible. Data processing: cryoSPARC v3.4, Topaz 0.2.4 and GraphPad Prism 9.2; Visualization: UCSF Chimera 1.15 and ChimeraX 1.2.5, PyMOL v2.6; Model building, refinement and validation: Coot 0.9.6, Phenix 1.20.1-4487, NAMDINATOR, 3DFSC; Secondary Structure: RNA2Drawer (RNACanvas), Adobe Illustrator

For manuscripts utilizing custom algorithms or software that are central to the research but not yet described in published literature, software must be made available to editors and reviewers. We strongly encourage code deposition in a community repository (e.g. GitHub). See the Nature Portfolio guidelines for submitting code & software for further information.

Data

Policy information about availability of data

All manuscripts must include a data availability statement. This statement should provide the following information, where applicable:

- Accession codes, unique identifiers, or web links for publicly available datasets
- A description of any restrictions on data availability
- For clinical datasets or third party data, please ensure that the statement adheres to our policy

All data are available in the main text and the supplementary materials. CryoEM maps generated in this study are deposited in the Electron Microscopy Data Bank with codes EMD-40986 (pre-1F), EMD-40985 (pre-2F), and EMD-40987 (post-2F), Structural models are available in the Protein Data Bank with PDB accession codes

8T2S (pre-1F), 8T2R (pre-2F), and 8T2T (post-2F). Spliceosome and group II intron models used for in this study (as an initial model for building or for comparison) are publicly available with the following PDB accession codes: 6J6Q (yeast spliceosome B* complex), 7B9V (yeast spliceosome C complex), 5MQ0 (yeast spliceosome C* complex), 7UIN (E.r. group II RNP), and 4FAQ (O.i. group II intron).

Research involving human participants, their data, or biological material

Policy information about studies with human participants or human data. See also policy information about sex, gender (identity/presentation), and sexual orientation and race, ethnicity and racism.

Reporting on sex and gender	N/A
Reporting on race, ethnicity, or other socially relevant groupings	N/A
Population characteristics	N/A
Recruitment	N/A
Ethics oversight	N/A

Note that full information on the approval of the study protocol must also be provided in the manuscript.

Field-specific reporting

Please select the one below that is the best fit for your research. If you are not sure, read the appropriate sections before making your selection.

☒ Life sciences ☐ Behavioural & social sciences ☐ Ecological, evolutionary & environmental sciences

For a reference copy of the document with all sections, see nature.com/documents/nr-reporting-summary-flat.pdf

Life sciences study design

All studies must disclose on these points even when the disclosure is negative.

Sample size	No statistical method was used to determine sample size. CryoEM sample sizes were determined by available microscope time and the particle density on electron microscopy grids. The sample size was sufficient to obtain structures at the reported resolution, as assessed by Fourier Shell Correlation (cutoff of 0.143). Four replicates were performed for the radioactive in vitro splicing assay with all mutant intron constructs and mutant maturase proteins. In vitro splicing time courses were done in duplicates for both conditions with and without the maturase protein.
Data exclusions	During cryoEM data processing, particles were excluded using standard classification approaches in cryoSPARC to remove false picks and low-resolution particle images. No data were excluded for the in vitro splicing assay.
Replication	All cryoEM structures were determined from independent half datasets, which were compared to determine the resolution of the respective reconstructions. It is not necessary to replicate cryoEM experiments and no replication was performed. The in vitro splicing assay with intron and maturase mutants was performed in four replicates and all attempts were successful and documented in the supplementary information. Time courses of intron splicing with and without the maturase protein were duplicated on different days with two different batches of radiolabeled RNA and independently purified maturase protein and yielded consistent banding patterns and rate constants.
Randomization	For cryoEM data processing, datasets are randomly split into two halves based on standard approaches in cryoSPARC. Randomization is not applicable for in vitro biochemical experiments.
Blinding	Blinding is not applicable to the in vitro biochemical or the cryoEM experimentation involved in this study.

Reporting for specific materials, systems and methods

We require information from authors about some types of materials, experimental systems and methods used in many studies. Here, indicate whether each material, system or method listed is relevant to your study. If you are not sure if a list item applies to your research, read the appropriate section before selecting a response.

Materials & experimental systems

n/a	Involved in the study
☒	☐ Antibodies
☒	☐ Eukaryotic cell lines
☒	☐ Palaeontology and archaeology
☒	☐ Animals and other organisms
☒	☐ Clinical data
☒	☐ Dual use research of concern
☒	☐ Plants

Methods

n/a	Involved in the study
☒	☐ ChIP-seq
☒	☐ Flow cytometry
☒	☐ MRI-based neuroimaging
